# 393. Characteristics of SARS-CoV-2 RNA Viral Loads among Nursing Home Residents and Staff with Repeat Positive Tests ≥ 90 Days After Initial Infection: 5 US Jurisdictions, July 2020–March 2021

**DOI:** 10.1093/ofid/ofab466.594

**Published:** 2021-12-04

**Authors:** W Wyatt Wilson, Kelly M Hatfield, Stacy Tressler, Cara Bicking Kinsey, Renee Zell, Channyn Williams, Kevin Spicer, Ishrat Kamal-Ahmed, Baha Abdalhamid, Mahlet Gemechu, Jennifer Folster, Natalie J Thornburg, Azaibi Tamin, Jennifer L Harcourt, Krista Queen, Suxiang Tong, Gemma Parra, John A Jernigan, John A Jernigan, Matthew B Crist, Kiran Perkins, Sujan Reddy

**Affiliations:** 1 Epidemic Intelligence Service, Centers for Disease Control and Prevention (CDC), Atlanta, Georgia; 2 Centers for Disease Control and Prevention, Atlanta, Georgia; 3 Bureau of Epidemiology, Pennsylvania Department of Health, Harrisburg, Pennsylvania; 4 Epidemiology and Surveillance Branch, DC Department of Health, Washington, District of Columbia; 5 Division of Epidemiology and Health Planning, Kentucky Department of Public Health, Frankfurt, Kentucky; 6 Division of Public Health, Nebraska Department of Health and Human Services, Lincoln, Nebraska; 7 Nebraska Public Health Laboratory, Omaha, Nebraska; 8 Division of Healthcare Quality Promotion, Centers for Disease Control and Prevention, Atlanta, Georgia; 9 Division of Viral Diseases, Centers for Disease Control and Prevention (CDC), Atlanta, Georgia; 10 Rollins School of Public Health, Emory University, Atlanta, Georgia

## Abstract

**Background:**

Background. Understanding the viral load and potential infectivity of individuals in nursing homes (NH) with repeat positive SARS-CoV-2 tests ≥ 90 days after initial infection has important implications for safety related to transmission in this high-risk setting.

**Methods:**

Methods. We collected epidemiologic data by reviewing records of a convenience sample of NH residents and staff with respiratory specimens who had positive SARS-CoV-2 rRT-PCR test results from July 2020 through March 2021 and had a SARS-CoV-2 infection diagnosed ≥ 90 days prior. No fully vaccinated individuals were included. Each contributed one repeat positive specimen ≥ 90 days after initial, which was sent to CDC and retested using rRT-PCR. Specimens were assessed for replication-competent virus in cell culture if Cycle threshold (Ct) < 34 and sequenced if Ct < 30. Using Ct values as a proxy for viral RNA load, specimens were categorized as high (Ct < 30) or low (if Ct ≥ 30 or rRT-PCR negative at retesting). Continuous variables were compared using Wilcoxon signed-rank tests. Proportions were compared using Chi-squared or Fisher’s exact tests.

**Results:**

Results. Of 64 unvaccinated individuals with specimens from 61 unique NHs, 14 (22%) were sent for culture and sequencing. Ten of 64 (16%) had a high viral RNA load, of which four (6%) were culture positive and none were known variants of interest or concern (Figure 1). Median days to repeat positive test result were 122 (Interquartile range (IQR): 103–229) and 201 (IQR: 139–254), respectively, for high versus low viral load specimens (p=0.13). More individuals with high viral loads (5/10, 50%) reported COVID-19 symptoms than with a low viral load (1/27, 4%, p=0.003). Most individuals (46/58, 79%) were tested following known or suspected exposures, with no significant differences between high and low viral load (p=0.18).

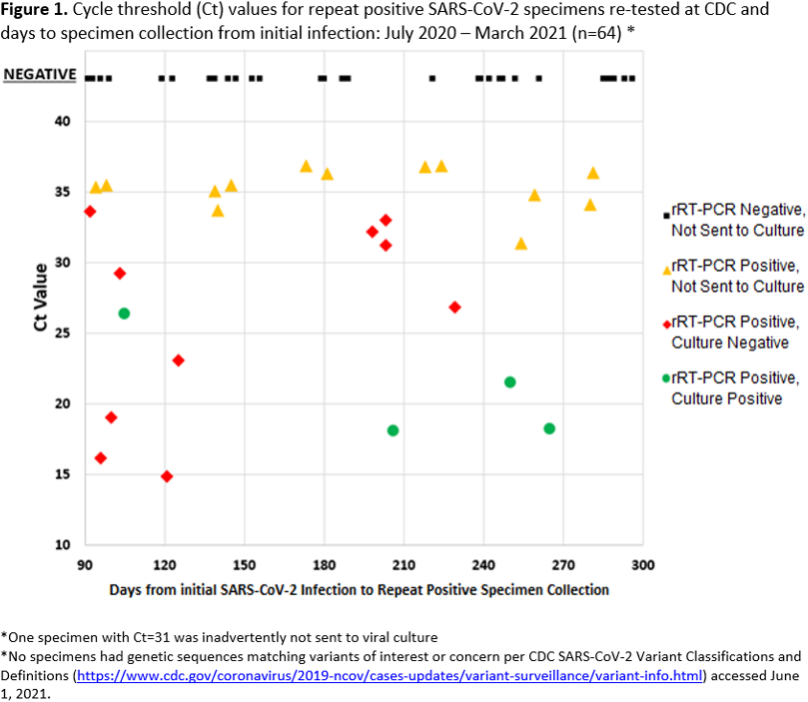

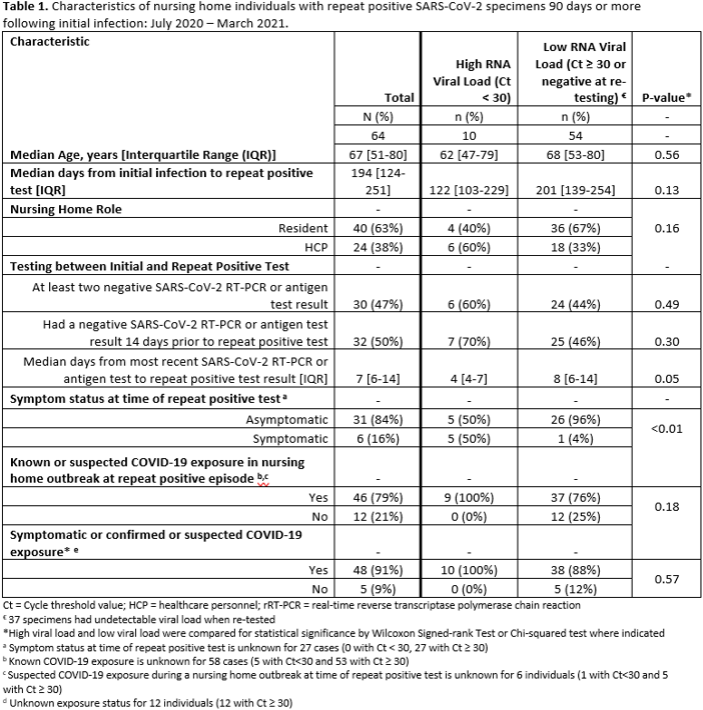

**Conclusion:**

In this study, nearly 1 in 6 NH residents and staff with repeat positive tests after 90 days demonstrated high viral RNA loads and viable virus, indicating possible infectivity. While individuals with high RNA viral load may be more likely to be symptomatic, distinguishing asymptomatic individuals who have high viral loads may be difficult with timing since initial infection, other test results, or exposure history alone.

**Disclosures:**

**John A. Jernigan, MD, MS**, Nothing to disclose.

